# Measuring person-centred care in german nursing homes – exploring the construct validity of the Dementia Policy Questionnaire: a cross-sectional study of a secondary data set

**DOI:** 10.1186/s12877-022-03586-3

**Published:** 2022-11-29

**Authors:** Anna Louisa Hoffman, Johannes Michael Bergmann, Anne Fahsold, René Müller-Widmer, Martina Roes, Bernhard Holle, Rebecca Palm

**Affiliations:** 1 Deutsches Zentrum für Neurodegenerative Erkrankungen (DZNE) e.V., Stockumer Str. 12, 58453 Witten, Germany; 2 Faculty of Health, Department of Nursing Science, Witten/Herdecke University (UW/H), Alfred- Herrhausen-Straße 50, 58448 Witten, Germany

**Keywords:** Nursing home, Person-centred care, Instrument development, Dementia care, Construct validity

## Abstract

**Background:**

To ensure the sustainable implementation of dementia-specific person-centred care (PCC) in nursing homes, internal policies are crucial. The preliminary German Dementia Policy Questionnaire, which features 19 dichotomous items, assesses the existence of and evaluates these policies. This article reports the results of an exploration of the construct validity of the preliminary Dementia Policy Questionnaire.

**Methods:**

This study is a cross-sectional study that references a secondary data set drawn from a national survey study of a randomized, stratified sample of 134 nursing homes in Germany. To explore the construct validity of the preliminary Dementia Policy Questionnaire, we conducted an adjusted multiple correspondence analysis of the pretested 19-item assessment. We included data assessed using the preliminary Dementia Policy Questionnaire from 134 care units associated with 134 nursing homes; these data were collected via telephone interviews with nursing home administrators or their representatives.

**Results:**

Two items assessing visitor regulations and regulations regarding the inclusion of residents in staff selection were less frequent and were therefore excluded from the adjusted multiple correspondence analysis. In total, nine items were assigned to two dimensions. The items assigned to the first dimension assess existing regulations for PCC as well as existing regulations regarding the involvement of the resident, relatives and the multiprofessional team in the collection of information concerning preferences, case conferences or decision making. The items assigned to the second dimension assess existing regulations regarding the systematic assessment of resident preferences and their requirements.

**Conclusion:**

The study produces exploratory evidence concerning the preliminary Dementia Policy Questionnaire. Since the dimensions of the items included in this questionnaire cannot be conceptualized clearly, the instrument in its current state requires further development.

## Background

This article introduces the preliminary German Dementia Policy Questionnaire (DemPol-Q), which is aimed at assessing the existence of internal policies regarding person-centred care in the context of dementia-specific care in nursing homes. The article describes the exploration of construct validity in this context by reference to a secondary data set.

In Germany, people with dementia are the largest group of residents in nursing homes [1]. In advanced stages of dementia, individuals experience agitation, anxiety, wandering, aggression, and psychosis more frequently [2, 3], a set of symptoms that are known as responsive behavior. To guarantee a good life for people with dementia, i.e., a life in which their physical and psychosocial needs are addressed simultaneously, residential long-term care requires best-practice models [4]. This necessity has resulted in a movement aimed at organizational cultural change [5] that focuses on the individual as well as his or her psychosocial needs [6]. Cultural change is driven by innovative care models, such as the person-centred approach [5, 7].

There are various definitions of person-centredness. The person-centred care (PCC) approach, which is aimed at all people who are in need of care, focuses on health care that is guided by the identified preferences and values of the people receiving the care [8]. To operationalize this approach in the context of nursing practice, Dewing, McCormack and McCance [9] developed the Person-centred Practice Framework, which includes (1) certain prerequisites, which refer to the competencies of nurses; (2) the nursing environment, which focuses on the nursing context; (3) person-centred processes, which focus on activities; and (4) the outcomes of effective PCC in practice.

Kitwood [10] distinguished the PCC approach in the context of dementia care from the standard medical approach. The aim of such care is to perpetuate the person’s identity [11], to appreciate the individuals’ personhood and to recognize the fact that the individual’s personality is concealed, not lost [4]. The delivery of dementia-specific PCC includes biographical knowledge, personalized surroundings, psychosocial environments, participation in social life, autonomy, and authentic relationship building [4, 12].

Dementia guidelines claim that dementia-specific care must be based on PCC [13]. Guidelines serve as relevant tools for implementing PCC and facilitate its sustainable implementation [13–15]. Nursing home providers are responsible for integrating the PCC approach into their mission statements and operationalizing the requirements mandated by these standards for their specific context of care. Such providers should develop and uphold evidence-based internal policies [16], which are necessary for staff with respect to their daily care practice [17]. These internal policies include written care standards, regulations or instructions.

In the United States, only 2% of nursing homes have implemented PCC approaches [5]. Missing internal policies are discussed as one factor inhibiting the implementation of PCC approaches but remain underinvestigated [17, 18]. To determine whether internal policies foster the implementation of PCC approaches, assessment is necessary. In Germany, the number of nursing homes that implement PCC approaches is unknown, and at present, no German assessment for the existence of internal policies regarding PCC in nursing homes that offer dementia-specific care has been developed.

We identified one assessment that evaluates the influence of internal policies on the person-centred management of responsive behavior in nursing homes from an organizational perspective: Resnick et al. [18] developed the “Assessment of Policies for Person-Centered Management of BPSD” based on both the expertise of the research group and certain policy factors discussed in the literature. This assessment includes 24 dichotomous items, 17 of which focus on policies regarding visiting hours, family/resident orientation and education, the collection of food preferences, the use of sensory aids, and safety rounds. Seven items regarding clinically relevant policies concentrate on patient-centred protocols for care planning, delirium, falls and the prevention of pressure ulcers. The total score of the assessment ranges from 0 to 24, and each item is identified as either existing or not. The higher the score is, the more effectively the internal policies in question contribute to facilitating the person-centred management of responsive behavior. The psychometric evaluation exhibited good reliability, including an internal consistency of 0.85 and an intraclass correlation of 0.88. Validity testing based on Rasch analysis indicated that the assessment was acceptable since nine items had high INFIT statistics, low OUTFIT statistics or high OUTFIT statistics.

The “Assessment of Policies for Person-Centered Management of BPSD” [18] provides a solid research foundation for evaluating the implementation of internal policies regarding the person-centred management of responsive behavior in the context of long-term residential care. We used this feasible assessment as a basis for developing an assessment for the German nursing home context.

### Development of the Dementia Policy Questionnaire (DemPol-Q)

To develop a preliminary German assessment, which we named the Dementia Policy Questionnaire (DemPol-Q), we used the “Assessment of Policies for Person-Centered Management of BPSD” by Resnick et al. [18] as a starting point. The development of the first version of the DemPol-Q involved three steps. (1) From November 2019 to February 2020, all items were reviewed by twelve scientific experts in dementia-specific long-term care. In accordance with the suggestions of Lynn [19], we asked these experts to rate each item in terms of its relevance to German nursing homes on a 4-point Likert scale (1 = very relevant; 4 = not relevant) and to comment on whether the item was adaptable to the German context. (2) RP and AF have expertise in residential long-term care and the necessary linguistic skills to carry out forward translation and cross-cultural adaptation in accordance with the suggestions of Maneesriwongul and Dixon [20]. They translated a total of 17 items since at least 80% of the experts considered these items to be cross-culturally adaptable and relevant to German nursing homes. In total, ten items matched our conception of recording resident preferences, while five items matched our notion of participatory decision making. Two items matched our conceptualization of dementia-specific interventions. (3) Because we considered nonpharmacological interventions beyond interventions aimed at the management of responsive behavior to be relevant to dementia-specific PCC, we developed an additional six items. These items follow the recommendations of the reviewed literature regarding interventions aimed at reducing psychotropic drug use to treat responsive behavior [2, 21–23]. Given that the DemPol-Q is designed to measure the existence of internal regulations regarding person-centred dementia care, the item format is dichotomous.

### Pilot testing of the DemPol-Q in german nursing homes

The first DemPol-Q draft featuring 23 dichotomous items underwent pilot testing by reference to a group of nursing home administrators and managers with experience working in dementia-specific care units. Four nursing homes provided their informed consent and participated in the pretest from March until May 2020. The first author collected data via computer-assisted telephone interviews. We asked participants to provide feedback regarding relevance, ease of answering, the existence of internal regulations, language and the comprehensibility of terms. We requested that they provide suggestions to develop more user-friendly wording. Based on the results of the pilot test, we adapted the language of items or increased their specificity. Three items that had been adapted from the assessment by Resnick et al. [18] and one literature-based item did not match the participants’ understanding of dementia-specific PCC in Germany. Since the participating nursing home administrators and managers had experience with dementia-specific long-term care and since we considered them to be experts in the field, we decided to exclude these items.

### DemPol-Q final draft

The final draft of the preliminary DemPol-Q contains a total of 19 dichotomous items. It aims to assess the existence of internal policies regarding dementia-specific PCC in German nursing homes. The total score of the measure ranges from 0 to 19, and items are identified as either existing or not.

Table 1 provides an overview of the preliminary DemPol-Q items, their categories, and their abbreviated names. This table also shows our a priori assignments of items to one of three parts: ‘Internal policies regarding the recording of residents’ preferences’, ‘Internal policies regarding participatory decision making’, and ‘Internal policies regarding dementia-specific interventions’. We based the names of the first and second parts of the preliminary DemPol-Q on aspects of person-centred processes (working with the person’s beliefs and values as well as shared decision making) based on the Person-centred Practice Framework [9].

The aim of this study was to explore the construct validity of the preliminary DemPol-Q.

**Table 1:** DemPol-Q items assigned to sections a priori, their categories, abbreviated names, and response frequencies

No.	Item	Categories	Abbreviated names	N_k_^a^ (*N* = 129)	F_k_^b^%
**Internal policies regarding the recording of residents’ preferences**					
Item 1	There are established visitor regulations that regulate visiting times and the number of visitors.	No	Visitor 0	132	98.5
		Yes	Visitor 1	2	1.5
Item 2	Preferences of residents are recorded systematically and in a structured way.	No	PreferenceA 0	24	17.9
		Yes	PreferenceA 1	110	82.1
Item 3	The hospitalization transfer form includes information regarding residents’ preferences.	No	Sheet 0	81	60.4
		Yes	Sheet 1	53	39.6
Item 4	There is a (written) procedure that includes residents in the staff selection process when employed.	No	Selection 0	132	98.5
		Yes	Selection 1	2	1.5
Item 5	There is a (written) procedure that specifies that residents or their legal representatives (relatives) are to participate in case conferences.	No	CConference 0	58	43.3
		Yes	CConference 1	76	56.7
Item 6	There is written policy regarding the manner in which nursing assistants are included in case conferences.	No	Involvement 0	65	48.5
		Yes	Involvement 1	69	51.5
Item 7	There is a written policy that provides residents with all-day use of common areas.	No	Area 0	39	29.1
		Yes	Area 1	95	70.9
Item 8	Residents’ preferences, which should be taken into account when performing prophylaxis, are recorded systematically and in a structured manner.	No	PreferenceB 0	30	22.4
		Yes	PreferenceB 1	104	77.6
Item 9	There is a policy regarding the manner in which external employees without regular access to nursing documentation (such as cafeteria service staff or external service providers) receive information concerning residents’ preferences.	No	PreferenceC 0	112	83.6
		Yes	PreferenceC 1	22	16.4
**Internal policies regarding participatory decision making**					
Item 10	There is a policy that stipulates that upon moving into the nursing home, a conversation regarding care planning is held with the resident and family members.	No	CarePlan 0	11	8.2
		Yes	CarePlan 1	123	91.8
Item 11	There is a policy that outlines the manner in which residents and family members are to be involved in procedures used as an alternative to restraint.	No	AltRestrict 0	34	25.4
		Yes	AltRestrict 1	100	74.6
Item 12	There is a policy regarding ways of dealing with refusals of nursing interventions and prophylaxis.	No	Rejection 0	53	39.6
		Yes	Rejection 1	81	60.4
**Internal policies regarding dementia-specific interventions**					
Item 13	The number of residents taking psychotropic drugs or neuroleptics is regularly evaluated as part of internal quality management.	No	Drugs 0	89	66.4
		Yes	Drugs 1	45	33.6
Item 14	Dementia-specific instruments are used to assess pain.	No	Pain 0	20	14.9
		Yes	Pain 1	114	85.1
Item 15	Dementia-specific behavioral assessment instruments are used.	No	Behavior 0	98	73.1
		Yes	Behavior 1	36	26.9
Item 16	Mandatory training on person-centred care is required for all staff.	No	Training 0	84	62.7
		Yes	Training 1	50	37.3
Item 17	One staff member is an expert in person-centred care (and both continuously educates himself or herself on this topic and supports others with respect to its implementation).	No	Expert 0	108	80.6
		Yes	Expert 1	26	19.4
Item 18	Dementia Care Mapping is conducted regularly (at least once per year) in the care unit by a person who does not work in the care unit.	No	DCM 0	116	86.6
		Yes	DCM 1	18	13.4
Item 19	Music therapy is offered at regular intervals (at least once per week) (prior to the COVID-19 pandemic) in the residential area by a trained music therapist.	No	Music 0	121	90.3
		Yes	Music 1	13	9.7

^a^absolute n_k_

^b^relative f_k_ in %

## Methods

### Study design

We conducted a cross-sectional study of a secondary data set associated with the national BeStaDem Survey. The BeStaDem Survey is an observational study featuring a cross-sectional design that includes a stratified randomized nationwide sample. The Deutsches Zentrum für Neurodegenerative Erkrankungen (DZNE) e.V., Site Witten, conducted the BeStaDem Survey with the aim of enhancing the typology of care units in German nursing homes.

### Setting

For the BeStaDem Survey, we recruited nursing homes from a commercially distributed list of all nursing homes in Germany [24], which was stratified in accordance with Germany’s 16 federal states and whether or not the nursing home in question included a Dementia Special Care Unit (DSCU). For each federal state, we planned to recruit ten nursing homes (eight without DSCU and two with DSCU). The inclusion criteria for the nursing homes included licensing as a nursing home with a reimbursement contract and the provision of all-day nursing care. We calculated the sample size based on the goal of recruiting an equal number of nursing homes per federal state as well as on the basis of feasibility. Using the stratified lists for each federal state, we contacted nursing homes in the order in which they were listed until we recruited ten nursing homes for each federal state.

### Participants

Since the purchased list provided contact information for every nursing home in Germany, we sent study information, including a declaration of consent, by mail to nursing home administrators working in the randomly selected nursing homes or their representatives. Subsequently, the first author contacted the nursing home administrators or their representatives (nursing home managers, care unit managers) by phone to ask whether they were interested in participating in the study. If so, they provided their written informed consent. We have reported additional details elsewhere [25]. The recruited sample includes a total of 134 nursing homes.

### Data collection

For the BeStaDem Survey, we recruited and collected data between June and December 2020. The first author, who was trained to conduct the interviews, collected data from the BeStaDem Survey via computer-assisted telephone interviews with an administrator or representative of the included nursing homes. For data collection, a standardized questionnaire was used that included the preliminary DemPol-Q. To prevent missing values, all items could be answered clearly and were easy to understand [25].

### Data sources

The BeStaDem Survey included a standardized questionnaire that inquired into the following:

- organizational characteristics at the nursing home level (full-time positions for nurses, the name and number of care units, the provision of dementia-specific care).
- organizational characteristics at the care unit level (architecture, financing, professionals, residents, meals, changes in structures resulting from the COVID-19 pandemic).
- the existence of internal policies regarding dementia-specific PCC in German nursing homes (DemPol-Q).
- sociodemographic and occupational information of the participants [25].

To describe the sample referenced by this study, we included three items regarding the type of care unit in question (DSCU/usual care unit), the form of provider (nonprofit/for profit), and the number of beds per care unit (mean). To explore construct validity, we included data from the preliminary DemPol-Q. The secondary data set was provided by the Deutsches Zentrum für Neurodegenerative Erkrankungen (DZNE) e.V., Site Witten.

### Statistical methods

On a theoretical basis, we assigned the items included in the DemPol-Q a priori to three parts without being aware of the correlations among them. To explore the construct validity of the instrument, we conducted a multiple correspondence analysis (MCA) [24, 26, 27] as an explorative analysis for data reduction. MCA is a form of principal component analysis used to explore categorical data [28]. This approach is common in the context of geometric mapping, where categories of items are assigned to dimensions that represent latent variables [26]. Since MCA underestimates the true quality of data representation, we conducted a modified procedure known as adjusted multiple correspondence analysis (adjusted MCA). Adjusted MCA improves the explained variation in the data per dimension [24].

Inertia is considered to be a measure of variance in the context of MCA. To facilitate the interpretation of the graphical mapping of the model, we calculated inertias for each dimension. Eigenvalues represent these inertias per dimension. The sum of the inertias of all dimensions defines the total inertia. The inertia of a dimension in relation to the total inertia indicates the percentage of inertia per dimension. These percentages indicate how well categories are represented as points on the respective dimension [27].

To discover a perfect low-dimensional space for our model, we included inertias per dimension, which provides a good explanation for the variation in data. As a decision rule, we conducted a scree test to identify a break in the curve as the cutoff criterion between inertias of dimensions in descending order. Inertias per dimension that were located prior to the break were included to facilitate interpretation.

For every category, we calculated coordinates that indicate a localization in the low-dimensional space. These localizations are characteristics of the categories with respect to the dimensions [26].

Each category has a relative inertia that contributes to the inertia of a dimension and determines the geometric direction of the dimensions in the low-dimensional space. Therefore, we calculated the contribution of the items and their respective categories to the dimension, which is denoted as Ctr. Items, and the categories that are informative with respect to a dimension are those whose contribution was above the baseline criterion (percentage value as the mean value) [27]. In accordance with the suggestions of Le Roux and Rouanet [27], we used contribution as the main aid for interpretation. To interpret the quality of the representation of single categories for a single dimension, we calculated the square of cosines for every category, which is defined as cos^2^. According to the recommendation of Blasius [26], we set a threshold for cos^2^ of 0.5, which corresponds to a cosine of 45°. Accordingly, the cosine of the angle between a category and a dimension must be at least less than 45° to ensure that a category is associated with a dimension. We used cos^2^ as an additive aid for orientation: when items and their categories in a dimension met both the contribution baseline criterion and the cos^2^ threshold of 0.5, we assigned these items to the corresponding dimension. If items and their categories in a dimension exhibited a very high contribution but the cos^2^ deviated slightly from the threshold of 0.5, we nevertheless assigned the item to the respective dimension if it seemed plausible in terms of content.

We conducted data analysis using the package ‘ca’ version 0.71.1 with the function ‘mjca’ [29] in R statistical software version 4.2.1 [30]. The MCA plot was generated using the package ‘ggplot2’ [31]. The data set and R code are available from the Zenodo repository [32].

We reviewed the data set drawn from the BeStaDem Survey with regard to plausibility and consistency prior to data analysis and identified no missing data.

### Ethical considerations

In October 2018, the ethics committee of the German Society of Nursing Science (Application number: 18 − 016) approved the study protocol for the BeStaDem Survey. To ensure data quality, we followed the relevant guidelines for good practice in secondary data analysis [33, 34].

## Results

### Participants and descriptive data

The stratified and randomized sample included in the data analysis for the adjusted MCA included 134 nursing homes from all 16 German federal states. Among the participating nursing homes, 56.7% were nonprofit organizations, and 43.3% were for-profit organizations. The sample included 79 nursing homes with usual care units and 55 nursing homes with DSCUs (Table 2).

**Table 2:** Comparison of sample characteristics between groups

Total Nursing Homes	*N* = 134 (100%)
**Care Unit Type: Usual Care Units**	*N* = 79 (59.0%)
**Care Unit Type: Dementia Special Care Units**	*N* = 55 (41.0%)
**Overall Provider Form**: Nonprofit including municipal and church organizations	*N* = 76 (56.7%)
**Overall Provider Form**: For-profit including private organizations	*N* = 58 (43.3%)
**Overall Number of Beds in the Care Unit (Mean)**	27.1

### Construct validity: results of the adjusted MCA

Table 1 indicates that both Item 1, which assesses visitor regulations, and Item 4, which assesses regulations regarding the inclusion of residents in staff selection, did not exist in all but two nursing homes. We excluded both items since less frequent categories contribute more to the total inertia and can cause biases in the adjusted MCA model.

The total inertia of the adjusted MCA model was 0.016094. Table 3 illustrates the eigenvalues, the proportion of explained inertia for the first eight dimensions of the model, and the scree plot that indicates a break between the second and the third inertia per dimension. We decided to include the first two dimensions for interpretation, as they contribute greatly to the explanation of inertia.

The first dimension ʎ_1_ explains 37.67% of total inertia, while the second dimension ʎ_2_ explains 21.92%. Together, these factors explain nearly 60% of the total inertia.

**Table 3:** Eigenvalues, proportion of explained inertia for the first eight dimensions, cumulative inertia, and scree plot

Dimension	Eigenvalues	Explained Inertia (%)	Cumulative Inertia (%)	Scree Plot
1	0.006063	37.67	37.67	**************
2	0.003528	21.92	59.59	********
3	0.000903	5.61	65.20	**
4	0.000308	1.91	67.12	*
5	0.000281	1.74	68.86	*
6	0.000028	0.17	69.03	
7	0.000013	0.08	69.12	
8	0.000001	0.00	69.12	
Total	0.016094	69.12		

All items with an average contribution of 100/17 = 5.88% to the dimension are informative and contribute significantly to the explanation of the included dimensions.

Table 4 shows that a total of eight items met the average item contribution of 5.88% for the first dimension. Among these items, five items and their respective categories met the cos^2^-determined threshold of 0.5. Since Item 6 and its respective categories exhibited a minor deviation from the cos^2^ threshold with a cos^2^ of 0.48 but made a high contribution of 12.92%, we included this item in the first dimension. Therefore, we assigned six items to the first dimension: Items 5, 6, 9, 11, 15, and 18.

**Table 4:** Assignment of items and categories to a dimension based on the adjusted MCA results

Item	Categories	Coordinates Dim 1	Coordinates Dim 2	Ctr (in %) Dim 1	Ctr (in %) Dim 2	Cos^2^ Dim 1	Cos^2^ Dim 2
**Items assigned to first dimension (Dim 1)**							
** Item 5**	CConference 0	-0.11	-0.03	4.68	0.81	0.50	0.05
	CConference 1	0.08	0.03	3.57	0.62	0.50	0.05
				Ctr item: 8.25	Ctr item: 1.43		
** Item 6**	Involvement 0	-0.12	-0.07	6.65	4.50	0.48	0.19
	Involvement 1	0.11	0.07	6.27	4.24	0.48	0.19
				Ctr item: 12.92	Ctr item: 8.74		
** Item 9**	PreferenceC 0	-0.04	-0.01	1.56	0.23	0.55	0.05
	PreferenceC 1	0.22	0.07	7.92	1.16	0.55	0.05
				Ctr item: 9.48	Ctr item: 1.39		
** Item 11**	AltRestrict 0	-0.19	-0.07	9.27	2.28	0.60	0.09
	AltRestrict 1	0.07	0.02	3.15	0.78	0.60	0.09
				Ctr item: 12.42	Ctr item: 3.06		
** Item 15**	Behavior 0	-0.08	0.03	4.20	0.86	0.68	0.08
	Behavior 1	0.21	-0.07	11.43	2.34	0.68	0.08
				Ctr item: 15.63	Ctr item: 3.20		
** Item 18**	DCM 0	-0.03	0.01	0.85	0.04	0.58	0.02
	DCM 1	0.20	-0.03	5.46	0.25	0.58	0.02
				Ctr item: 6.31	Ctr item: 0.29		
**Items assigned to second dimension (Dim 2)**							
** Item 2**	PreferenceA 0	-0.05	0.25	0.36	18.45	0.02	0.58
	PreferenceA 1	0.01	-0.05	0.08	4.03	0.02	0.58
				Ctr item: 0.44	Ctr item: 22.48		
** Item 8**	PreferenceB 0	-0.07	0.19	1.18	13.68	0.07	0.47
	PreferenceB 1	0.02	-0.06	0.34	3.95	0.07	0.47
				Ctr item: 1.52	Ctr item: 17.63		
** Item 16**	Training 0	-0.01	0.07	0.07	5.00	0.01	0.49
	Training 1	0.02	-0.12	0.12	8.40	0.01	0.49
				Ctr item: 0.19	Ctr item: 13.40		
**No assignment possible**							
** Item 3**	Sheet 0	-0.05	0.04	1.64	1.51	0.40	0.21
	Sheet 1	0.08	-0.06	2.51	2.31	0.40	0.21
				Ctr item: 4.15	Ctr item: 3.82		
** Item 7**	Area 0	-0.10	0.04	2.64	0.64	0.34	0.05
	Area 1	0.04	-0.01	1.08	0.26	0.34	0.05
				Ctr item: 3.72	Ctr item: 0.90		
** Item 10**	CarePlan 0	-0.25	-0.13	4.90	2.49	0.35	0.10
	CarePlan 1	0.02	0.01	0.44	0.22	0.35	0.10
				Ctr item: 5.34	Ctr item: 2.71		
** Item 12**	Rejection 0	-0.11	-0.09	4.64	4.97	0.43	0.27
	Rejection 1	0.07	0.06	3.04	3.25	0.43	0.27
				Ctr item: 7.68	Ctr item: 8.22		
** Item 13**	Drugs 0	-0.03	0.00	0.43	0.00	0.40	0.00
	Drugs 1	0.05	0.00	0.85	0.00	0.40	0.00
				Ctr item: 1.28	Ctr item: 0.00		
** Item 14**	Pain 0	-0.15	0.15	3.21	5.54	0.38	0.38
	Pain 1	0.03	-0.03	0.56	0.97	0.38	0.38
				Ctr item: 3.77	Ctr item: 6.51		
** Item 17**	Expert 0	-0.04	0.03	1.19	1.10	0.42	0.22
	Expert 1	0.16	-0.12	4.95	4.57	0.42	0.22
				Ctr item: 6.14	Ctr item: 5.67		
** Item 19**	Music 0	-0.01	0.01	0.07	0.05	0.11	0.04
	Music 1	0.09	-0.05	0.68	0.49	0.11	0.04
				Ctr item: 0.75	Ctr item: 0.54		

The six items summarize opposing categories that identify to a reasonable degree whether internal policies exist. While the negative side of the first dimension includes categories concerning the absence of such policies, the positive side of the first dimension includes categories pertaining to the existence of internal policies regarding PCC. The first dimension also reveals an ordinal order for the existing regulations for PCC: sections far away from the center of the positive section particularly include categories of regulations for dementia-specific interventions such as regular DCM (Item 18) and the use of dementia-specific behavioral assessments (Item 15). The categories that are shown close to the center of the positive section of the first dimension instead include items pertaining to the involvement of the resident, relatives, and the multiprofessional team in the collection of information regarding preferences, case conferences or decision making.

**Fig. 1:**
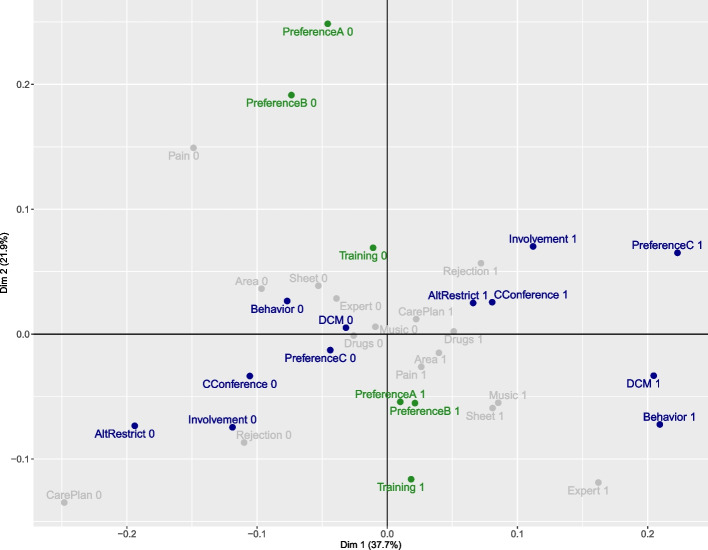
Adjusted MCA Plot.  Item categories assigned to the first dimension.  Item categories assigned to the second dimension.  Item categories that cannot be assigned to a dimension

With respect to the second dimension, five items met the baseline criterion, for a contribution of 5.88% (Table 4). The items pertaining to the assessment of residents’ preferences (Item 2), the assessment of preferences regarding the performance of prophylaxis (Item 8), and the provision of mandatory training on PCC to staff (Item 16) make the largest contribution to the inertia of the second dimension. To the categories of Item 2, which meet the cos^2^ determined threshold of 0.5, we add the categories of Item 8, which feature a cos^2^ of 0.47, and those of Item 16, which feature a cos^2^ of 0.49, with minor deviations from the determined threshold, since they also make large contributions. Therefore, we assigned Items 2, 8, and 16 to the second dimension. According to Fig. 1, the coordinates of categories and their algebraic sign categories provide a good summary of a contrast: categories related to the absence of internal policies are assigned to the positive section of the second dimension, while categories related to the presence of internal policies are assigned to the negative section of the second dimension. We also identified an ordinal order for the second dimension. “Mandatory training on PCC” on the negative section, which is located far from the center, can be viewed as the “PCC requirement for systematic assessment of resident preferences”, which is located near the center.

The numerical interpretation is consistent with Fig. 1: categories that have low distances from each other are positively correlated, while binary categories are located opposite to each other and negatively correlated. Eight gray items with their respective categories are poorly represented by or make small contributions to the first and second dimensions. These categories lie between several dimensions and cannot be assigned clearly.

## Discussion

This study aimed to explore the construct validity of the preliminary DemPol-Q. The adjusted MCA showed that nine items were significantly assigned to two dimensions.

Items 1 and 4, which assessed internal policies regarding visitor regulations and the inclusion of residents in staff selection, exhibited very low frequencies and were thus excluded from the adjusted MCA. In the “Assessment of Policies for Person-Centered Management of BPSD” [18], which indicated strong support for both item reliability (coefficient alpha 0.85) and interrater reliability (ICC 0.88), these items could be distinguished well and were associated with good INFIT and OUTFIT statistics regarding construct validity. The data referenced in our study illustrate that 98.5% of nursing homes allow relatives to visit residents at any time, but 98.5% indicated that they exclude residents from staff selection due to challenges in recruiting new staff. We removed items 1 and 4 from the preliminary DemPol-Q since they are not relevant to the topic of PCC in German nursing homes.

The items assigned to the first dimension clarify two different aspects of dementia-specific PCC. Since the preliminary DemPol-Q comprises items 5, 6, 9, and 11, which were adapted from the assessment by Resnick et al. [18], their content reflects the inclusion of residents and relatives in the interdisciplinary work of residents as cases. The comparable items developed by Resnick et al. [18] exhibited good INFIT and OUTFIT statistics, with the exception of comparable Item 6 (INFIT 2.0, OUTFIT 2.05) that includes policies regarding the involvement of nursing assistance in three areas (care planning versus performance improvement activities versus involvement in shift report). The DemPol-Q items assessing regular DCM (Item 18) and the use of behavioral assessments (Item 15) were assigned a priori to the part ‘Internal policies for dementia-specific interventions’ and are now assigned to the first dimension. The rationale for this assignment is the recommendation of nonpharmacological interventions as the preferred choice in the management of responsive behavior [2, 35].

Items 2 and 8, which are now assigned to the second dimension, were assigned a priori to the part ‘Internal policies for recording residents’ preferences’. Item 16 correlates with items 2 and 8 and exhibits an ordinal order. This finding is in line with the results of other studies. Chenoweth et al. (2015) found that staff education is viewed as a priority for the implementation of PCC, while intervention studies or protocols that aim to measure the effectiveness of PCC in nursing homes combine the implementation of PCC interventions with preliminary PCC training [36–38].

Eight items could not be assigned to the included dimensions. As shown in Fig. 1, Item 14, which assesses dementia-specific pain instruments, fits exactly between the first and second dimensions. Item 14 appears to be related to both dimensions since both nursing homes that assess preferences and nursing homes that involve the resident, relatives, and the multiprofessional team in the collection of information regarding preferences, case conferences or decision making reported using dementia-specific instruments to assess pain. All eight items define another construct that is accounted for by no other variable included in the DemPol-Q, and all eight are defined equivocally, misunderstood or have no relevance to dementia-specific PCC in Germany. Regarding Item 13, which assesses the evaluation of residents’ psychotropic drug use by quality management, participants may not have known how to respond since affirmation requires quality management for evaluation. Comparable items in the assessment conducted by Resnick et al. [18] exhibited acceptable INFIT and OUTFIT statistics.

The adjusted MCA results indicate that the assignment of items to the two dimensions in question corresponds only partially to the a priori assignments of the preliminary DemPol-Q, in which context the first two parts were denominated in accordance with aspects of the Person-centred Practice Framework [9]. In our results, items are assigned in accordance with the manner in which they are implemented by German nursing homes. With regard to the first dimension, some nursing homes frequently mandate internal policies regarding DCM (Item 18) and often issue internal policies concerning the use of behavioral assessments (Item 15). It can thus be suggested that the items adapted from the assessment by Resnick et al. [18] may not correspond in terms of content to the specific understanding of dementia-specific PCC in the context of German nursing homes. Therefore, we decided not to denominate the first and second dimensions as latent variables. The assignment of categories to dimensions cannot yet be designated unambiguously by reference to a specific subdimension of dementia-specific PCC. Therefore, we maintain that the current version of the DemPol-Q requires further development.

Since most of the items were adapted and translated from Resnick et al. [18], these items measure internal policies aimed at the management of responsive behavior and represent only some aspects of dementia-specific PCC. According to Kitwood [10] and the requirements stipulated by international guidelines [13], the DemPol-Q does not contain any items for measuring internal policies, e.g., autonomy, authentic relationship building, or psychosocial environments. Kitwoods’ approach is specific to people with dementia. The DemPol-Q aims to serve as a way of evaluating the existence of internal policies regarding dementia-specific PCC in the context of long-term care as well as a means of providing statements concerning the different degrees to which dementia-specific PCC is present in various dementia-specific long-term care settings. Further development of the DemPol-Q in collaboration with professionals working in the fields of nursing research and practice who have expertise regarding dementia-specific PCC in Germany is necessary in the following contexts: (1) discussing the appropriateness of a dichotomous response scale and reconsidering the type of scale used for the DemPol-Q; (2) discussing statistical test theory and considering item response theory methods to measure the degree to which internal policies regarding dementia-specific PCC are present in nursing homes; (3) enhancing items that were not specifically assigned to the included dimensions based on the results of the adjusted MCA; and (4) discussing the dimensionality of the DemPol-Q to develop additional items and identify its latent variables clearly.

The results of this study indicate that aspects of person-centred care are described in the internal policies of nursing homes in Germany. Consequently, we concluded that internal regulations can contribute to the provision of good quality of care and thus to the sustainable implementation of person-centred care. Other studies have discussed internal policies as instruments for facilitating or hindering person-centred care [39]. For internal policies to be significantly beneficial for staff, these regulations must find a balance according to which staff can comply with the policies while simultaneously providing dementia-specific person-centred care [40]. This task includes the incorporation of knowledge regarding nursing practice [41] and the needs of staff into such policies [40].

To explore the construct validity of the preliminary DemPol-Q, we used a secondary data set associated with the BeStaDem Survey, which focuses on a stratified, randomized, nationwide sample of 134 nursing homes that is representative of Germany. Although we evaluated a data set that was valid and appropriate, the answers of study participants during the telephone interviews might have been biased. Some of the items included in the DemPol-Q inquire into particular topics, for example, the policy of using dementia-specific instruments to assess pain (which is mandatory in nursing homes in Germany), but the answers of the study participants might have been influenced by social desirability bias if they had implemented an internal policy requiring the use of such an assessment tool.

For statistics, we employed adjusted MCA as a modified procedure for MCA. This approach is considered to be an exploratory data reduction procedure that remains untested in the context of nursing science by comparison to principal component analysis. In this study, we demonstrated that adjusted MCA is an appropriate way of intelligibly representing the data structures of categorical data in low-dimensional space.

## Conclusion

Further research is necessary to identify (1) which internal policies regarding dementia-specific PCC exist and to what degree as well as which policies are most relevant in this context and (2) ways of integrating key aspects of dementia-specific PCC into internal policies in German nursing homes. On the basis of this evidence, it is possible to enhance the preliminary DemPol-Q to reflect the progress of organizational culture change and that of the process by which dementia-specific PCC is implemented in nursing homes.

## Data Availability

The data set generated and/or analyzed for the current study and the R code are available in the Zenodo repository, DOI 10.5281/zenodo.6912951.

## Abbreviations

Cos^2^: Square of Cosine
Ctr: Contribution
DemPol-Q: Dementia Policy Questionnaire
DSCU: Dementia Special Care Unit
MCA: Multiple Correspondence Analysis
PCC: Person-Centred Care
