# Correction: Measuring person-centred care in German nursing homes – exploring the construct validity of the Dementia Policy Questionnaire: a cross-sectional study of a secondary data set

**DOI:** 10.1186/s12877-022-03724-x

**Published:** 2023-02-13

**Authors:** Anna Louisa Hoffmann, Johannes Michael Bergmann, Anne Fahsold, René Müller-Widmer, Martina Roes, Bernhard Holle, Rebecca Palm

**Affiliations:** 1 Deutsches Zentrum für Neurodegenerative Erkrankungen (DZNE) e.V., Stockumer Str. 12, 58453 Witten, Germany; 2 Faculty of Health, Department of Nursing Science, Witten/Herdecke University (UW/H), Alfred-Herrhausen-Straße 50, 58448 Witten, Germany

**Correction: BMC Geriatr 22, 914 (2022)**

**https://doi.org/10.1186/s12877-022-03586-3**

After publication of this article [1], the authors reported that the author name ‘Hoffmann’ was incorrectly written as ‘Hoffman’. Moreover, In the Results section citations to references [24] and [25] were mixed up.

The original article [1] has been corrected.
